# Curative Effect of Cutaneous Ulcer Wound Repair Through a Novel Liquid Dressing After HpD‐PDT Treatment of Extramammary Paget's Disease—A Prospective, Semi‐Lesion Controlled, Pilot Study

**DOI:** 10.1111/jocd.70421

**Published:** 2025-09-03

**Authors:** Dixin Wang, Yan Zhao, Qihang Chang, Peiru Wang, Guolong Zhang, Xiuli Wang

**Affiliations:** ^1^ Institute of Photomedicine, Shanghai Skin Disease Hospital, Tongji University School of Medicine Shanghai China

## Abstract

**Objective:**

To evaluate the clinical efficacy and safety of a novel skin wound dressing in promoting the repair of the ulcer wound after the treatment of Extramammary Paget's Disease (EMPD) with Hematoporphyrin Derivative Photodynamic Therapy (HpD‐PDT).

**Method:**

A total of 10 patients with EMPD previously treated with HpD‐PDT were recruited to conduct a self semi‐lesion controlled study on the treatment of conformal dressing to evaluate the efficacy and safety of the skin ulcer wound repair therapy after HpD‐PDT. The skin lesions treated with novel HVHA Shutai dressing were the study intervention group, and the skin lesions treated with standard of care were the control group. Parameters including skin lesion wound area and adverse events were collected and analyzed in both groups, and wound healing rates were calculated.

**Results:**

The mean total healing time of 10 subjects was 14.80 ± 3.36 weeks on the study intervention side and 18.30 ± 4.16 weeks on the control side. The total healing time of the study intervention side was significantly less than that of the control side (*p* = 0.009). The average healing rate of the test side was 85.28% ± 17.15% at Week 12, and that of the control side was 70.48% ± 23.20% at Week 12. The average healing rate of the study intervention side was significantly higher than that of the control side at Week 12 (*p* = 0.004). All skin lesions were completely healed at the end of follow‐up. No adverse events such as dressing allergy, pain, or wound infection occurred in the subjects.

**Conclusion:**

For skin wound care after HPD‐PDT treatment of EMPD, HVHA Shutai dressing can accelerate wound healing, promote skin tissue and cell regeneration, and significantly shorten the time for complete wound healing of skin lesions, with significant effectiveness and acceptable safety.

## 1 Introduction

Extramammary Paget disease (EMPD) is a rare intraepidermal cancer. Disease onset ages generally range from 50 to 80 years old. The lesions often occur in the apocrine gland distribution, locating on the vulva, scrotum, penis, perianal area, groin, armpit, and other places, mostly as a single lesion, but they can also occur as multi‐focal lesions. Most lesions are eczematoid patches and may be accompanied by pain, itching, late ulceration, bleeding, nodules, and sclerosis. Invasive EMPD invading the dermis has a high metastasis rate and poor prognosis. With the intensification of the aging society, the incidence of EMPD is increasing year by year. Surgical treatment is the primary treatment for EMPD, but it is prone to recurrence; large trauma and special site, as well as a large area of skin grafts and reconstruction, often face problems such as postoperative skin appearance and function impairment, limiting treatment benefits. Previously, our team has successfully applied HiPorfin hematoporphyrin derivatives photodynamic therapy (HpD‐PDT) to treat EMPD after the approval by the ethics committee of our hospital, which has advantages of deep skin lesion treatment, repeatable treatment, small trauma, and protection of the structure and tissue function preservation of some specific locations, and is effective and practical. However, due to the large wound surface of patients after HpD‐PDT treatment and the probable secondary infection after treatment, there is a huge unmet need in clinical practice for a novel treatment for anti‐infection, wound care, and repair after HpD‐PDT treatment of extramammary Paget disease.

Dressings for skin lesion wounds include HVHA Shutai silver ion antibacterial agent, HVHA Shutai silver ion antibacterial gel, bacterial cellulose biological application, and elastic bandage dressing. In this study, 10 patients with EMPD who were treated with HpD‐PDT were recruited to conduct a self‐controlled study on the treatment of conformal dressing to evaluate the efficacy and safety of the skin ulcer wound repair therapy after HpD‐PDT.

## 2 Method

This study was approved by the Ethics Committee of Shanghai Skin Disease Hospital. Informed consent was obtained from all participants before their involvement. All procedures were conducted in accordance with the ethical standards of the Declaration of Helsinki and Good Clinical Practice guidelines.

HpD‐PDT procedure: Patients received intravenous injection of the photosensitizer Hiporfin (Hematoporphyrin Derivative, HpD, Chongqing Maile Bio‐pharmaceutical Liability Company) at a dose of 3.0 to 5.0 mg/kg body weight. After 48 h, lesions were irradiated using a 630‐nm diode laser (Guilin Xingda Photoelectric Medical Instrument Co. Ltd., Guilin, China) at a light dose of 150 to 200 J/cm^2^. The irradiation time was calculated based on lesion area and light power density. All PDT procedures were performed in a dedicated photodynamic therapy suite at Shanghai Skin Disease Hospital.

Self‐control method was used. The skin lesions treated with novel HVHA Shutai dressing were the interventional group and the skin lesions treated with standard of care (SoC) were the control group.

Study intervention group: for study intervention side, the wound was wiped and debrided with the HVHA Shutai silver ion antibacterial agent; HVHA Shutai silver ion antibacterial gel coating; HVHA Shutai bacterial cellulose dressing for external application; finally, the wound was secured with elastic bandage dressing.

Control group (SoC): for the control side, the wound surface was debrided with boric acid, a wet compress with ethacridine lactate solution for 20 min was applied, fusidic acid and vaseline gauze were applied externally, and then fixed with gauze.

Dressing changes were performed daily for both the study intervention and control groups.

Scheduled visits: define the date of the last photodynamic therapy for the subject prior to study commencement as Day 0. Follow‐up was performed at 1, 3, and 7 days and 1, 2, 4, 8, and 12 weeks after treatment (if the ulcer wound healed completely within 12 weeks, treatment was stopped, the healing time was recorded and the wound condition was evaluated; if the ulcer wound did not heal after 12 weeks, treatment was continued, and follow‐up was performed once after complete healing).

Effectiveness evaluation method: the total effective rate was calculated for each group. The area and depth of lesions were measured, and the adverse reactions (pain, pruritus, infection, erythema, etc.) were evaluated.

Efficacy and safety evaluation are described below:

### 2.1 Primary Endpoints

#### 2.1.1 Wound Healing Rate

The wound healing of patients before and after treatment was observed, and the wound healing rate was calculated according to the wound healing area measured at different time points (before treatment, 14 ± 2 days after treatment, 28 ± 2 days, 56 ± 3 days after treatment).

$$\text{The wound healing rate}=\frac{\left(\text{wound area before treatment}-\text{wound area after treatment}\right)}{\text{wound area before treatment}}\times 100\%$$

### 2.2 Secondary Endpoints

1. Total healing time: Duration from first dressing application to complete wound closure (weeks).
2. Therapeutic efficacy categories (assessed at Week 12):
  - Obvious effect: > 40% area reduction, significant exudate decrease, fresh new granulation without edema.
  - Effective: 20%–40% area reduction, the exudate was reduced, the wound margin was softened, and the new granulation tissue was red without edema.
  - Ineffective: < 20% reduction or enlargement. The exudate was not significantly reduced, with granulation tissue aging.

### 2.3 Safety Endpoint

Adverse events (AEs) and serious adverse events (SAEs): All AEs/SAEs were recorded from informed consent to study completion per ICH‐GCP guidelines. AEs of special interest including dressing allergy, pain, infection, erythema, and pruritus would be actively monitored throughout the study period.

#### 2.3.1 Inclusion Criteria

1. Pathological diagnosis of EMPD, age ≥ 18 years old, male or female;
2. Treatment with HpD‐PDT;
3. Voluntarily participate in a clinical trial when other alternatives have been informed, are willing to pay for treatment, and agree to have the lesion photographed;
4. No serious hematopoietic coagulation function and heart, lung, liver, kidney function abnormalities or immune deficiency;
5. After fully explaining the clinical trial drug and the purpose and content of the clinical trial (including the subject's compliance, etc.), voluntarily sign the informed consent to participate in the clinical trial on the basis of full understanding.

#### 2.3.2 Exclusion Criteria

1. Known allergy to the experimental drug or related drugs;
2. During the treatment period of this study, other wound repair treatments were received simultaneously;
3. Participate in any drug clinical trial (as a subject) within 1 month before participating in this trial;
4. Neutrophil counts < 1.5 × 10^9^/L, platelets < 100 × 10^9^/L, or hemoglobin < 100 g/L; serum creatinine was 3 times higher than the upper limit of the normal reference range or creatinine clearance was < 60 mL/min/1.73 m^2^; ALT or AST > 3 times the upper limit of normal; ALT or AST > 5 times the upper limit of normal in cases of liver metastasis; serum bilirubin was 3 times higher than the upper limit of the normal reference range.
5. Complicated with serious diseases, including serious heart disease, cerebrovascular disease, uncontrolled diabetes, uncontrolled hypertension, etc.;
6. Subjects infected with HIV, HCV, syphilis, or hepatitis B surface antigen and e antigen are positive;
7. Subjects with acute inflammation or clinically significant active infection;
8. Pregnant or lactating women, and participants of reproductive age (including men) who refused to use appropriate contraception during the study period;
9. The subject is in a state of cachexia, or the assessment of advanced subjects cannot tolerate this treatment;
10. Subjects with a serious physical or mental illness that the investigator believes may affect treatment, evaluation, or poor adherence to the study protocol.

The experimental drugs were provided by Jiangsu Changshu HVHA Medical Technology Development Co. Ltd., and the previous treatment (photodynamic therapy drugs, light source, etc.) and the control group treatment was provided by the department of photodynamic therapy, Shanghai Skin Disease Hospital.

## 3 Results

A total of 10 subjects with EMPD who had undergone HpD‐PDT were enrolled. All subjects included in the study completed treatment and follow‐up. Subject baseline condition details could be found in Table 1; among them, 9 were male and 1 was female. The average age of subjects was 73.20 ± 6.80 years old, and the average thickness of lesions was 3.15 ± 2.21 mm. The mean wound area at baseline on the study intervention side (the first day after photodynamic therapy, defined as Day 1) was 28.21 ± 18.24 cm^2^, and the mean wound area on the control side at baseline was 38.53 ± 28.06 cm^2^. The mean lesion area of the control group was numerically larger than that of the study intervention group but without statistical significance (*p* > 0.05).

**TABLE 1:** Baseline subject condition.

Subject ID	Gender	Age (yo)	Disease course (yrs)	Lesion location	Lesion thickness (mm)	Initial lesion area of study treatment (cm^2^, Day 1)	Initial lesion area of control treatment (cm^2^, Day 1)
1	M	79	2	Vulva	2.3	21.54	51.02
2	M	70	2	Vulva	0.88	15.56	18.53
3	M	68	2	Vulva	4.2	14.53	18.78
4	F	63	10	Vulva	0.7	13.25	15.12
5	M	70	3+	Perianal	1.2	9.35	10.80
6	M	76	5+	Vulva	2	66.30	58.21
7	M	71	3+	Vulva	2.5	28.80	31.06
8	M	81	3+	Axilla	0.9	33.85	37.74
9	M	85	10+	Vulva	1.7	51.41	104.69
10	M	69	6	Right vulva	1.5	27.55	39.33

**FIGURE 1:**
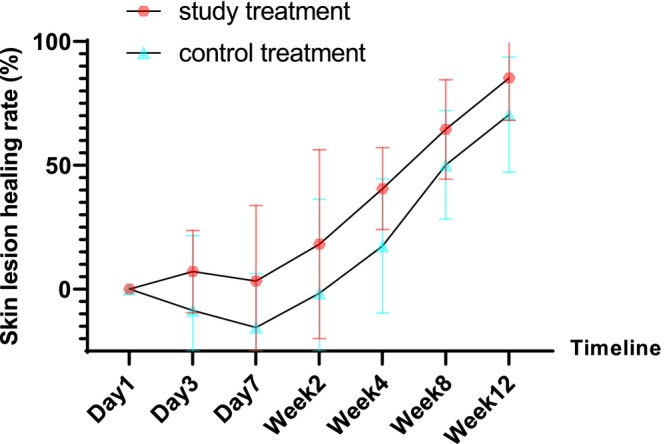
Mean skin lesion healing rate of EMPD subjects before and after bilateral treatment.

**FIGURE 2:**
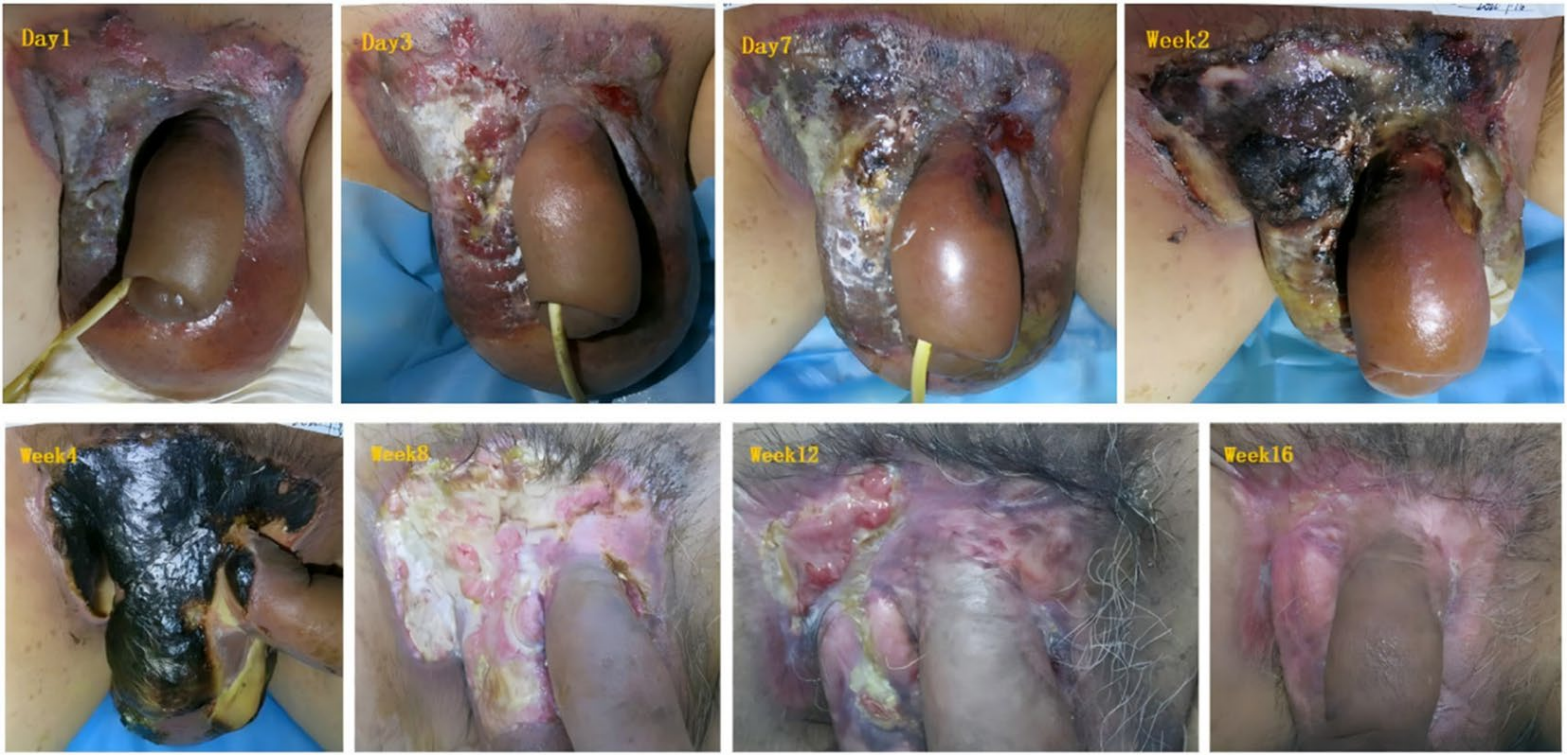
Lesion conditions at both sides of EMPD subjects after skin wound care (the right side of the subject is the control side, the left side is the study intervention side).

Table 2 recorded change of skin lesion area on both sides of subjects and final healing time. The mean total healing time of 10 subjects was 14.80 ± 3.36 weeks on the study intervention side and 18.30 ± 4.16 weeks on the control side. The total healing time of the study intervention side was significantly less than that of the control side (*p* = 0.009). The average healing rate of the test side was 85.28% ± 17.15% at Week 12, and that of the control side was 70.48% ± 23.20% at Week 12. The average healing rate of the study intervention side was significantly higher than that of the control side at Week 12 (*p* = 0.004). All skin lesions were completely healed at the end of follow‐up. Table 3 recorded the distribution of different therapeutic effects on the study intervention side and the control side at the 12th week, and the proportion of lesion complete response on the study intervention side of the subjects (100%) was higher than that on the control side (80%). Figure 1 recorded the change of the average skin wound healing rate of EMPD subjects before and after bilateral treatment. It can be seen that the skin healing status of subjects on the study intervention side was significantly better than that on the control side at each time point. Figure 2 shows the skin lesions on both sides of a subject after wound care (the right side of the subject is the control side and the left side is the study intervention side). It can be intuitively reflected in the photos that the skin wound areas on the study intervention side shrink faster and heal earlier at around 2 months after the beginning of wound care treatment.

**TABLE 2:** Changes of subject skin lesion area and final healing time.

Subject ID (study treatment/control treatment)	Lesion area, Day 3 (cm^2^)	Lesion area, Day 7 (cm^2^)	Lesion area, Week 2 (cm^2^)	Lesion area, Week 4 (cm^2^)	Lesion area, Week 8 (cm^2^)	Lesion area, Week 12 (cm^2^)	Complete healing period (weeks)
1 (Study treatment)	18.05	16.81	9.78	9.10	7.23	8.13	16
1 (Control treatment)	52.45	58.13	42.48	57.47	26.58	32.43	18
2 (Study treatment)	10.30	10.57	27.86	12.34	10.52	7.17	15
2 (Control treatment)	19.95	19.38	35.43	21.20	16.48	9.63	18
3 (Study treatment)	14.63	13.95	12.33	6.82	0	0	8
3 (Control treatment)	20.70	22.41	23.51	10.07	2.66	0	12
4 (Study treatment)	11.72	11.25	8.14	6.70	2.57	0	12
4 (Control treatment)	14.56	12.83	11.95	9.65	6.21	4.41	21
5 (Study treatment)	8.15	11.66	7.99	5.03	6.06	2.62	15
5 (Control treatment)	10.85	13.31	8.36	5.15	7.31	6.86	24
6 (Study treatment)	73.64	58.49	67.09	52.19	25.21	9.51	14
6 (Control treatment)	71.06	71.34	68.83	50.47	31.15	10.86	14
7 (Study treatment)	25.37	23.03	21.69	11.61	10.71	4.68	20
7 (Control treatment)	26.45	38.03	35.69	16.89	16.35	9.93	25
8 (Study treatment)	42.56	57.84	20.29	17.19	7.38	0.16	18
8 (Control treatment)	70.61	61.26	33.28	35.66	13.55	5.45	19
9 (Study treatment)	41.86	51.55	38.03	43.51	18.11	1.63	17
9 (Control treatment)	82.38	90.61	63.77	82.96	26.40	3.63	17
10 (Study treatment)	26.51	21.02	14.20	18.39	10.26	0.31	13
10 (Control treatment)	37.67	45.13	30.45	46.66	26.26	7.24	15

**TABLE 3:** Distribution of different efficacy cases on the study intervention side and the control side at Week 12.

Dressing treatment efficacy	Obvious effect	Effective	Ineffective	Lesion complete response percentage ((obvious effect cases)/total cases*100%)
Numbers of study intervention group	10	0	0	100
Numbers of control group	8	2	0	80

No AE or SAE such as dressing allergy, pain, or wound infection was reported in any subject.

## 4 Discussion

Extramammary Paget's Disease (EMPD) is an intraepithelial cancer that occurs predominantly in the distribution area of sweat glands and can occur in the vulva, scrotum, penis, perianal, groin, and armpits. Surgical excision is the preferred method of treatment, but it has the disadvantages of easy recurrence, large trauma, and affected appearance and function of those involved in flap transplantation or reconstruction. Current disadvantages of the surgical treatment of EMPD include the risk of recurrence due to difficulties in some surgery regions, like lesions that are too large to be operated on or lesions located in multiple skinfold areas, which is not an indication for surgical excision.

Photodynamic therapy (PDT) is a treatment based on the administration of a topical or systemic photosensitizer, which is distributed to the lesions, and then a light source with a specific wavelength enters the lesions to selectively destroy the target tissue, sparing healthy tissue [1, 2]. Besides, PDT is a non‐invasive method that is simple and practical, which can be repeatedapplied. Nowadays, PDT has been reported to be able to effectively treat diseases such as condyloma acuminatum, severe acne, and squamous cell carcinoma, as well as employed in EMPD [3, 4, 5, 6]. According to previous study on HpD‐PDT for EMPD treatment, intravenous or systemic HpD‐PDT is able to offer acceptable disease outcomes including relatively acceptable complete response (CR) rate, similar recurrence without distinct cosmetic and functional impairment [3]. When the innovative HpD‐PDT could be promising in EMPD treatment, cutaneous wound healing remains a complex and dynamic physiological process during which damaged tissues are repaired and skin integrity is restored, thus photodynamic therapy still brings the remaining problem of wound healing as surgery does. In some patients, it is difficult to achieve wound healing, and a long recovery period is needed to obtain closure [7].

Our study mainly investigated the clinical effectiveness and safety of HVHA shutai dressing for ulcer wound repair after HpD‐PDT in the treatment of EMPD. The total healing time of the study side was significantly less than that of the control side. The skin lesions of all subjects were completely healed at the end of follow‐up, and the area of the skin lesions on the study intervention side was reduced more quickly, and the complete healing period was shorter, reflecting better efficacy. During our study treatment, potential adverse reactions such as dressing allergy, pain, and wound infection were closely observed, and no adverse events or serious adverse events occurred in the subjects. It is proposed that the ideal wound dressings should have the characteristics of non‐toxicity, retaining moisture, and promoting wound healing [8]. The positive effect of the use of wound dressings on the healing of skin lesions may be achieved through more than one mechanism. The porous structure of the dressing is conducive to the timely discharge of wound exudate and the prevention of infection. Moreover, the coverage of the wound by the dressing can reduce the invasion of microorganisms and prevent skin wound infection. A key factor which may contribute to the positive wound healing results in the study is considered to be the wound wet healing effect through the dressings. Based on previous landmark studies, it was shown that wounds exposed to air to dry tend to heal more slowly with poor cosmesis when compared to wound healing with moisture [9, 10]. Various studies supported the benefits from moist wound healing with positive outcomes for healing achieved in a variety of wound types when wound dressing was designed to provide optimal hydration level [11]. The dressing in our research delivered a good environment for wound healing, especially a proper moist environment.

The limited sample size and lack of long‐term safety follow‐up of the study restrict the study conclusion expansion. Although silver ions (Ag^+^) exhibit efficient and broad‐spectrum antibacterial activity, their potential cytotoxicity in the body caused by their uncontrolled and direct release profile cannot be ignored; thus, more long‐term studies focusing on safety and metal ion‐related adverse reactions are needed in the future.

## 5 Conclusion

In summary, for the skin wound care after HPD‐PDT treatment of EMPD, HVHA Shutai dressing can accelerate wound healing, promote skin tissue and cell regeneration, and significantly shorten the time for complete wound healing of skin lesions, with significant effectiveness and acceptable safety, indicating an inspiring prospect for clinical use. The conclusions of this study are limited by sample size and need to be further verified with a larger sample size and a longer follow‐up period.

## Author Contributions

D.W. and X.W. designed the research study. D.W. and Y.Z. performed the experiments and collected data. D.W., Q.C., and G.Z. analyzed the data. D.W. wrote the manuscript. P.W., G.Z., and X.W. contributed to critical revisions. X.W. supervised the project and provided essential reagents. All authors (D.W., Y.Z., Q.C., P.W., G.Z., X.W.) read and approved the final manuscript.

## Conflicts of Interest

The authors declare no conflicts of interest.

## Data Availability

Research data are not shared.
